# Why Should Constant Stimulation of Saccular Afferents Modify the Posture and Gait of Patients with Bilateral Vestibular Dysfunction? The Saccular Substitution Hypothesis

**DOI:** 10.3390/jcm11041132

**Published:** 2022-02-21

**Authors:** Ian S. Curthoys, Paul F. Smith, Angel Ramos de Miguel

**Affiliations:** 1 Vestibular Research Laboratory, School of Psychology, The University of Sydney, Sydney, NSW 2006, Australia; 2 Department of Pharmacology and Toxicology, School of Biomedical Sciences, University of Otago, Dunedin 9016, New Zealand; paul.smith@otago.ac.nz; 3 The Brain Health Research Centre, University of Otago, Dunedin 9016, New Zealand; 4 Department of Otolaryngology, and Head and Neck Surgery, Complejo Hospitalario Universitario Insular Materno Infantil de Gran Canaria, 35016 Las Palmas de Gran Canaria, Spain; aramos.gcc@gmail.com

**Keywords:** bilateral vestibular dysfunction, saccular, posture, gait, vestibular implant, otolith

## Abstract

An ongoing EU Horizon 2020 Project called BionicVEST is investigating the effect of constant electrical stimulation (ES) of the inferior vestibular nerve in patients with bilateral vestibular dysfunction (BVD). The evidence is that constant ES results in improved postural stability and gait performance, and so the question of central importance concerns how constant ES of mainly saccular afferents in these BVD patients could cause this improved performance. We suggest that the constant ES substitutes for the absent saccular neural input to the vestibular nuclei and the cerebellum in these BVD patients and indirectly via these structures to other structures, which have been of great recent interest in motor control. One target area, the anterior midline cerebellum (the uvula), has recently been targeted as a location for deep-brain stimulation in human patients to improve postural stability and gait. There are projections from midline cerebellum to basal ganglia, including the striatum, which are structures involved in the initiation of gait. It may be that the effect of this activation of peripheral saccular afferent neurons is analogous to the effect of deep-brain stimulation (DBS) by electrodes in basal ganglia acting to help alleviate the symptoms of patients with Parkinson’s disease.

## 1. Introduction

Patients with bilateral vestibular dysfunction (BVD) [1] show postural instability and gait difficulties. One treatment for BVD that is under investigation in a multicentre EU project (called BionicVEST) is to implant a long-term stimulating electrode on one inferior vestibular nerve and deliver a constant train of high-frequency electrical pulses by that electrode (1200 biphasic pulses per second continuously) and measure the behavioural and perceptual effects of that electrical stimulation (ES). The electrode location is such that the ES is probably mainly stimulating saccular afferents. Patients are selected according to strict criteria to meet the Barany guidelines for BVD [2]. As of 3 January 2022 a total of 10 patients has been implanted.

Preliminary results continue to be obtained, some of which have been published [1]. It is clear that this constant electrical stimulation acts to improve the patient’s postural stability and gait performance as measured by Computerized Dynamic Posturography (CDP) and the Dynamic Gait Index (DGI). There are also changes in a number of tests of peripheral vestibular function, which are described in detail in [1]. The patients report that this constant stimulation is beneficial, and they do not want it switched off. With this stimulus, they find they have less need for assistance in walking and stabilising themselves.

Here, the aim is to state a simple hypothesis about why this constant, high-frequency electrical stimulation could have these effects and to review the evidence relevant to that hypothesis. The hypothesis is called the “saccular substitution hypothesis,” and it is that the neural activity generated by the ES is substituting for the reduced or absent constant saccular afferent activity from the saccular macula. We reviewed recent evidence relevant to that hypothesis about the role of direct and indirect vestibular input for postural stability and gait performance.

The saccular stimulation activates the descending spinal pathways as well. It may be that the effect of this saccular stimulation is analogous to the effect of deep-brain stimulation (DBS) by electrodes in the basal ganglia acting to help alleviate the symptoms of patients with Parkinson’s disease. Possibly, the constant stimulation of the saccular afferents in the vestibular nerve is just a step further back: so, rather than directly stimulating the basal ganglia electrically, now, it is a matter of stimulating saccular afferents electrically, which project via the cerebellum to the basal ganglia and also to the spinal cord and so result in changes in performance.

BionicVEST is a pilot programme to investigate the effect of constant electrical stimulation (ES) of inferior vestibular nerve in patients with BVD [1] according to the consensus criteria of the Barany Society [2]. The aim is to determine if this constant ES results in changes in performance on:

- Tests of peripheral vestibular function,
- Tests of postural stability and gait performance, and
- Patient subjective experience. Do they find constant ES to be an improvement?

## 2. The Steps in the Research Process

1. Selection. Patients are selected according to carefully defined selection criteria defining bilateral vestibular dysfunction (BVD). It should be stressed that there is some residual peripheral vestibular function in these patients, but their level of function falls below the international accepted standard for bilateral vestibular dysfunction (Barany Society consensus statement [2]);
2. Preoperative testing of peripheral vestibular function, postural stability and gait (e.g., using the DGI and CDP, etc.);
3. The electrode and implantation. A modified cochlear implant electrode is implanted on a branch of the vestibular nerve in one ear. The exact location of the electrode depends on particular anatomical considerations at surgery. The aim is to implant the electrode very close to the inferior vestibular nerve containing afferents from the saccular macula and posterior semicircular canal. From the CT verified electrode locations, it appears that the electrode locations so far have been in the inferior vestibular nerve and so largely otolithic, mainly saccular afferents. It is likely there is some stimulation of the afferents from the posterior canal; however, it is necessary to emphasise that this is uncertain. We will refer to the data as being due to saccular stimulation, whereas in fact, it more likely should be referred to as vestibular stimulation (probably mainly saccular);
4. Post-operative testing. Initially, it is necessary to verify that the stimulating electrode is functional and make adjustments of the stimulus parameters of the ES (relying on patient subjective reports of oscillopsia or subjective sensations);
5. The stimulus. Then, the ES is switched on a constant train of pulses at high-frequency (900 or 1200 pulses/s), and it is kept on continuously. It is important to stress that with this implant, there is, unlike other vestibular implants [3], no modulation of the pulse rate or pulse amplitude in response to the movement of the patient. The ES is composed of a constant pulse train delivered by three electrodes of a modified cochlear implant electrode. Each pulse is biphasic (25 microseconds per phase) and delivered at a frequency of 900 or 1200 pulses per second. Three electrodes are activated, so the stimulus is simply a constant train of pulses, which will activate vestibular afferents at a very high rate, presumably duplicating the constant barrage of action potentials that healthy patients receive;
6. Stimulation phase. The patient is then re-tested on repeated occasions in the postoperative phase (in some patients, this phase has lasted 1.5 years so far). On each testing occasion, this is done first to ensure the electrode is working. Fibrosis or changes in impedance may make the electrode non-functional, in which case vestibular and clinical tests would be rendered meaningless. Then, on each occasion, they are tested on the battery of tests of peripheral vestibular function, postural stability, gait performance, and acceptability. Their results on these tests are then compared to their preoperative measures and their performance in comparison to the electrode OFF condition and measures of patient satisfaction (usually relying on patient subjective reports or oscillopsia).

## 3. Preliminary Test Results

See Ramos Macias et al. [1] for detailed results. On a priori grounds, we did not expect some vestibular tests to show changes: for example, we did not expect ES of saccular afferents to affect semicircular canal function (as measured by VOR gain using the video Head Impulse Test (vHIT)). However, it was possible that measures of otolith function could change as a result of the ES stimulation. We were particularly concerned to identify if there were improvements in tests of postural stability and gait performance.

## 4. The Saccular Substitution Hypothesis

With this saccular ES, patients show improved postural stability and gait performance (e.g., measured by the DGI or CDP), and so the question of central importance consists of how constant electrical stimulation of saccular afferents in these BVD patients could cause this improved performance. We consider that this maintained stimulation is generating a barrage of action potentials in the saccular nerve, which is substituting for the reduced or absent saccular afferent action potentials in these BVD patients. The saccular macula is unique in that saccular receptors are continuously stimulated by the force of gravity. There are a large number of primary saccular afferents (around 4000 in humans, [4,5]) and these saccular afferents have high resting discharge rates (probably around 50–100 spikes/s in humans [6]), so each second, a huge barrage of action potentials is continuously reaching the vestibular nuclei and the midline cerebellum and, we suggest, indirectly projecting on to other structures, e.g., the basal ganglia. Bilateral vestibular loss will reduce or remove this sustained neural input, and it appears that the high-frequency electrical stimulation by the implanted vestibular electrode is substituting for this lost natural neural activation.

Hence, their loss is depriving their target nuclei of a huge amount of neural input, which is presumably a major contributing factor to the poor performance of BVD patients on posture and gait tasks pre-operatively.

The questions then become:

1. Where do saccular afferents project to? and
2. How could their sustained activation influence posture and gait performance?

To address these questions, we present a brief overview of evidence about direct and indirect saccular projections and refer to the effect of damage to these target areas on patient performance. There is new evidence about how deep-brain stimulation acts upon some of these target areas to improve patient gait and postural stability. In the following sections, we consider the effects of ES of the vestibular nuclei, basal ganglia, the cerebellum, and the pedunculopontine nucleus (PPN) of the mesencephalic locomotor region in patients with deficits of motor control for improving postural stability and gait performance.

## 5. Saccular Projections

As is clear from the anatomical evidence of the direct and indirect central projections of saccular afferents, their neural activity has very widespread effects in motor control systems. Saccular afferents project to brainstem vestibular nuclei, to spinal cord, and to cerebellum and indirectly through the cerebellum to the basal ganglia and to other structures, which have been of great recent interest in motor control, especially the pedunculopontine nucleus (PPN) of the mesencephalic locomotor region (MLR). Therefore, the activation of saccular afferents is indirectly activating many motor control systems.

**Figure 1:**
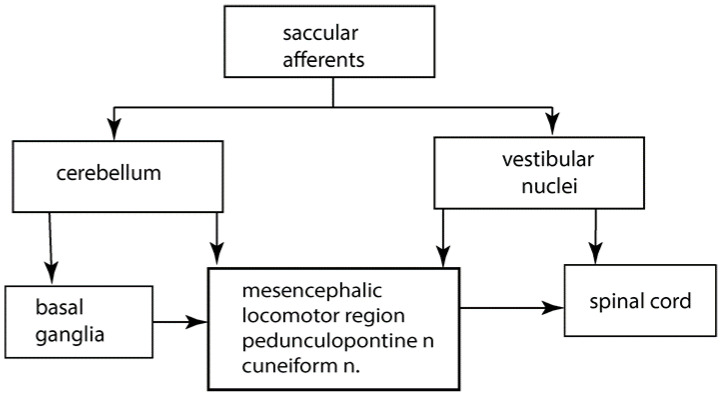
A greatly simplified schematic overview of some of the major structures involved in the control of gait and postural stability.

Saccular afferents travel in the inferior vestibular nerve, along with afferents from the posterior semicircular canal. Saccular afferent fibres branch and send a thinner collateral branch to the cerebellum, with the thicker afferent branch projecting to the vestibular nuclei. Saccular fibres terminate in the lateral and inferior vestibular nuclei. Anatomical evidence shows that saccular afferents project both to vestibular nuclei and the cerebellum, specifically to the anterior midline structures, including the uvula [7,8,9] and deep cerebellar nuclei [10] (see Figure 1).

## 6. The Role of the Cerebellum in Posture and Gait

There is extensive evidence of the crucial role of the cerebellum in the control of balance and locomotion (see, e.g., [11]). Cerebellar circuits connect with many brain and spinal cord nuclei. Cerebellar activity is required for motor behaviours ranging from coordination to posture and balance and gait. This is clear from the deficits in these behaviours in human patients with localized cerebellar damage [11]. Projections from the deep cerebellar nuclei influence basal ganglia activity by afferents that project to the thalamic nuclei, which project to the basal ganglia primarily the striatum [11]. Dijkstra reported the cerebellum is involved in postural control as shown by image analysis [12]. Mori et al. [13] demonstrated in cats that stimulation of the midline cerebellar locomotor region (the fastigial nucleus) can independently induce locomotion. Neuroimaging suggests a similar region exists in humans. Studies with mental imagery of gait or foot pedals showed that active stepping during fMRI causes focal increases in the fastigial nucleus of the cerebellum and cerebellar vermis [12]. The cuneiform nucleus also appears to be of interest.

All the studies suggested the cerebellum is implicated in all aspects of the pathophysiology of Parkinson’s disease. One of the most characteristic signs of cerebellar damage is walking ataxia. Anterior cerebellar damage (to the so-called vestibulo cerebellum) leads to increased postural sway [11,14,15]. Cerebellar damage is also associated with hypermetric postural responses to surface displacements and impaired ability to learn responses to predictable perturbations or step initiation [16]. Some of these deficits that are described for patients with cerebellar loss appear to be similar to those described for patients with bilateral vestibular loss.

It is not known exactly how the cerebellum normally contributes to walking although recent work suggests that it plays a role in the generation of appropriate patterns of limb movements, dynamic regulation of balance, and adaptation of posture and locomotion through practice [17,18]. Patients with cerebellar loss show excessive or diminished responses to perturbations, with poor control of equilibrium during motion and abnormal oscillation of the trunk. Gait ataxia is often described with distinctive features, including variable foot placement, irregular foot trajectories, wide base of support, a veering path of movement, and abnormal inter-joint coordination [11]. These are similar to the gait disturbances of patients with BVD [1].

## 7. Cerebellar Locomotor Region

The cerebellar locomotor region is in the midline of the cerebellum [19], and as noted above, it is this region where saccular afferents terminate. Neurons from the central cerebellum project back to the vestibular nuclei [4,7,8,9,10,20,21,22]. Physiological studies from animals suggest that cerebellar control of posture, equilibrium, and locomotion are tightly controlled and localised in the medial zone. There are cerebellar projections to vestibular and reticular nuclei and also to the thalamus. Thus, the medial cerebellar zone can integrate spinal and vestibular inputs and influence motor pathways for walking.

## 8. Cerebellum to Basal Ganglia

Manto elaborated on the short latency connections between the cerebellum and the basal ganglia [23,24,25]. These connections may explain the cerebellar involvement in disorders commonly associated with basal ganglia dysfunction, for example, Parkinson’s disease. Recently, cerebellar neurons are being stimulated in human patients by brain stimulation techniques, including transcranial magnetic stimulation and transcranial direct current stimulation, to alleviate disturbances of motor control [13,26,27,28,29]. In an animal model, Miterko reported that neuromodulation of the cerebellum rescues movement in a mouse model of ataxia [26].

## 9. The Pedunculopontine Nucleus (PPN)

The mesencephalic locomotor region (MLR) consists of the pedunculopontine tegmental nucleus (PPN), the cuneiform nucleus, and the sub-cuneiform nucleus. It receives input from cerebellar nuclei and basal ganglia [30,31,32]. Imaging of the PPN during imagined walking shows that it is involved in control of postural stability [33,34,35], and electrical stimulation of PPN is a target for treating locomotion deficits [36].

## 10. Vestibular Nuclei to Basal Ganglia

The most convincing evidence that there may be disynaptic projections from the vestibular nuclei to the basal ganglia was published by Lai et al. [37]. Using neuronal tracers, they reported that projections from the medial vestibular nucleus to the parafascicular nucleus (PFN) of the thalamus synapse on neurons that project to the dorsolateral putamen of the striatum. This anatomical evidence suggested the possibility of a disynaptic pathway between the vestibular nuclei and the striatum albeit from the medial vestibular nucleus rather than the inferior and lateral vestibular nuclei, where saccular afferents are known to terminate. The PFN is also strongly connected to the PPN [38]. Although there have been a small number of electrophysiological studies investigating whether electrical stimulation of the peripheral vestibular system can evoke field potentials and single-unit activity in the striatum (see [38,39] for reviews), none of these can exclude the possibility that any responses arise via the cerebellum, and none of them are involved selective saccular stimulation. Nonetheless, there are many regions of the striatum that have not been explored, such the striatal tail, which is known to receive substantial visual and auditory sensory input and may also receive vestibular input (see [40] for a review). Whether saccular information is transmitted to this multisensory integration centre remains to be determined but seems very likely.

## 11. Gait

Normal gait is a complex process that involves concomitant balance and locomotion processes. A hierarchy of supraspinal regions send signals to the central pattern generators (CPGs) of the spinal cord [35,41]. Supraspinal regions modify stereotyped locomotion in certain situations, such as initiating gait, turning, stopping, and avoiding obstacles. The locomotor network involves CPGs, mesencephalic locomotor region, the cerebellar locomotor areas, subthalamic locomotor region, and various cortical areas, including frontal and parietal supplementary motor and motor areas [35].

## 12. Contribution of the Otoliths to Spatial Awareness

Part of any qualitative improvement in BVD patients following ES may be attributable to its effects on higher centres of the brain concerned with the cognitive processing of vestibular information, especially spatial awareness and memory (“spatial cognition”). The effects of inferior vestibular nerve ES on spatial cognition have not been investigated quantitatively in humans; therefore, the only evidence available is from rodents.

There has been increasing evidence that the otoliths, independently of the semi-circular canals, may be important for spatial cognition. It is difficult to surgically manipulate the saccule in animal models without affecting the other vestibular sensors. Therefore, the only evidence available is restricted to mutant mice, which do not generate otoconia (e.g., *tilted Het* and *Otop* mice; see [39] for a review). Inevitably, this means that the mice are devoid of both utricular and saccular function rather than just saccular function. However, due to its role in the perception of gravity, the saccule might be expected to be particularly important in providing a gravitational reference frame for other sensory information. A recent study in rats reported that selective electrical stimulation of the saccule resulted in widespread activation of the bilateral hippocampus, a structure that is important for spatial memory [42]. Quantitative gait analysis has not been performed on these otolith-deficient mouse models as yet; however, they do exhibit substantial deficits on the rotarod, in exploration and in performance in Y maze, radial arm maze, elevated plus maze, and place recognition tasks (see [39] for a review). These results suggest that loss of otolithic function, including saccular function, results in deficits in spatial cognition. Studies of the first 10 days of development indicate that *Het* mice develop abnormally, exhibiting abnormal responses in the righting reflex, cliff drop aversion, and negative geotaxis tests [43]. There is also evidence that thalamic head direction cells and hippocampal place cells function abnormally in otolith-deficient mice (see [39] for a review). Taken together, these studies suggest that the saccule is important for spatial awareness and orientation and that saccular ES may have a beneficial effect in BVD patients.

## 13. Conclusions

This review has shown the widespread direct and indirect projection of saccular afferent neural activity to brain structures controlling postural stability and gait performance. In patients with BVD, this neural activity will be reduced or absent, and we suggest that the constant ES is activating primary saccular afferents and hence is substituting for the absent saccular afferent neural activity, therefore acting to improve postural stability and gait. 

## Data Availability

Not applicable.
